# Encapsulation of Human Umbilical Cord Mesenchymal Stem Cells in LunaGel Photocrosslinkable Extracellular Matrix and Subcutaneous Transplantation in Mice

**DOI:** 10.3390/biomedicines11041158

**Published:** 2023-04-12

**Authors:** Truc Le-Buu Pham, Dang Phu-Hai Nguyen, Thao Thi-Thu Luu, Luong Si Nguyen, Nguyen Trong Binh, Quan Dang Nguyen, Phong Anh Tran

**Affiliations:** 1 Biotechnology Center of Ho Chi Minh City, Ho Chi Minh City 700000, Vietnam; plbtruc.snn@tphcm.gov.vn (T.L.-B.P.);; 2 Histology-Embryology-Pathology Department, Faculty of Medicine, University of Medicine and Pharmacy at Ho Chi Minh City, Ho Chi Minh City 700000, Vietnam; 3 Interface Science and Materials Engineering Group, School of Mechanical, Medical and Process Engineering, Faculty of Engineering, Queensland University of Technology, Brisbane City, QLD 4000, Australia

**Keywords:** LunaGel, cell-laden scaffold, mesenchymal stem cells, regenerative medicine, umbilical cord

## Abstract

Stem cells have significant potential in regenerative medicines. However, a major issue with implanting stem cells in the regeneration of new tissue is the methods to implant them and cell viability and functions before and after implantation. Here we developed a simple yet effective method that used photo-crosslinkable gelatin-based hydrogel (LunaGel^TM^) as a scaffold for the encapsulation, expansion, and eventually, transplantation of human umbilical cord-derived mesenchymal stem cells (hUC-MSCs) into mice subcutaneously. We demonstrated the proliferation and maintenance of the original expression of mesenchymal stem cell markers as well as the ability to differentiate into mesoderm-derived cells. The hydrogel was highly stable with no signs of degradation after 20 days in PBS. The hUC-MSCs remained viable after transplantation into mice’s subcutaneous pockets and migrated to integrate with the surrounding tissues. We showed a collagen-rich layer surrounding the transplanted cell-laden scaffold indicating the effects of growth factors secreted by the hUC-MSCs. A connective tissue layer was found between the implanted cell-laden scaffold and the collagen layer, and immunohistochemical staining results suggested that this tissue was derived from the MSCs which migrated from within the scaffold. The results, thus, also suggested a protective effect the scaffold has on the encapsulated cells from the antibodies and cytotoxic cells of the host immune system.

## 1. Introduction

In 1993, the concept of tissue engineering was described by Langer and Vacanti as “an interdisciplinary field that applies the principles of engineering and the life sciences toward the development of biological substitutes that restore, maintain, or improve tissue function” [1]. There have been a number of achievements in using tissue engineering approach to treat damaged or diseased tissues [2,3,4]. The three main components in tissue engineering are the cells, the scaffolds for carrying the cells, and the physicochemical factors of the environment [5].

Cells used in tissue engineering are usually autologous progenitors or stem cells. In recent years, the research and use of mesenchymal stem cells in tissue engineering have attracted increasing attention [6,7,8]. Mesenchymal stem cells were first isolated in 1970 [9]; they have a number of desirable properties, such as self-renewal, the capability to be differentiated into different cell types, and immunomodulatory capabilities [10]. The immunomodulatory properties of mesenchymal stem cells would minimize the chance of causing the host’s immune responses compared to other cell types [11,12]. Importantly, mesenchymal stem cells can be obtained non- or minimum-invasively from various sources such as bone marrow, adipose tissue, and umbilical cords [13].

Mesenchymal stem cells obtained from autologous sources, such as adipose tissue or bone marrow, are limited in number (depending on the condition, age, and cloning time before transplanting) [14]. Therefore, our search team focused on mesenchymal stem cells derived from umbilical cord tissue, which is abundantly available with no risk of viruses or other harmful agents. The isolation of mesenchymal stem cells from umbilical cord tissue was also well-established [15].

In terms of scaffolds for encapsulating and transplanting cells in tissue engineering applications, the scaffolds should be biocompatible and provide an environment to support the functions of encapsulated cells [16]. Hydrogels are networks of cross-linked polymers with high water content. Being highly porous, hydrogels support cell attachment and growth [17]; having high water content encourages the exchange of ions, nutrients, and metabolites with the fluids of the surrounding environment to support the cells that are encapsulated within the hydrogels [18].

LunaGel™ is a photo-crosslinkable extracellular matrix (ECM) based on chemically modified pharmaceutical-grade gelatin. The hydrogel is formed when a photo-initiator is added to the sterile solution of the photo-crosslinkable ECM and irradiated with 405 nm light. The hydrogel’s main components include the ECM protein collagen types I, III, IV, and V, as well as connective tissue glycoproteins and proteoglycans. The hydrogel LunaGel ™ maintains cells’ biological activity and facilitates cell cohesion, proliferation, differentiation, and migration.

In this study, we investigated the application of LunaGel as the hydrogel scaffold for the encapsulation and subcutaneous transplantation of human umbilical cord-derived mesenchymal stem cells (hUC-MSCs), with an ultimate aim of using this as a tissue engineering therapy in reconstructive surgeries.

## 2. Materials and Methods

### 2.1. Isolation and Culture of Umbilical Cord-Derived Mesenchymal Stem Cell

Human umbilical cord tissues were provided by the MekoStem Stem Cell Bank (Ho Chi Minh City, Vietnam). Human umbilical cord-derived mesenchymal stem cells (hUC-MSCs) were isolated following the method of Yan-Fu Han et al. [19]. The hUC-MSCs were cultured in MSC NutriStem^®^ XF Medium (Biological Industries, Cromwell, CT, USA) at 37 °C in 5% CO_2_.

### 2.2. Criteria for Assessing the Characteristics of hUC-MSCs

The characteristics of the isolated hUC-MSCs were evaluated in passage 5. The cell’s surface receptors were assessed by flow cytometry for the following markers: CD105; CD73; CD90; CD45; CD34; CD11b; CD19; and HLA-DR, using the Human MSC Analysis Kit (BD Biosciences, San Jose, CA, USA). The candidate cells were differentiated into osteoblasts, adipocytes, and chondrocytes in vitro, using MSCgo ™ Rapid Osteogenic Differentiation Medium (Biological Industries), MSCgo ™ Adipogenic Differentiation Medium (Biological Industries), and MSCgo ™ Chondrogenic Differentiation Medium (Biological Industries), respectively. After 21 days, the differentiated cells (osteoblasts, adipocytes, and chondrocytes) were stained with Alizarin Red, Oil Red, and Alcian Blue, respectively. The images were digitally recorded under a light microscope (Nikon-TS2). The collected hUC-MSCs were also checked for mycoplasma using the MycoAlertTM Plus Mycoplasma Detection kit (Lonza, Basel, Switzerland).

### 2.3. Preparation of Hydrogel Scaffold

Photo-crosslinkable Extracellular Matrix (LunaGel^TM^, Gelomics, Australia) was supplied as a sterile solution. It was mixed with the supplied freeze-dried photo-initiator, which was previously reconstituted in PBS, poured into the mold, and cross-linked by irradiating with 405 nm light (LunaCrosslinker^TM^) according to the manufacturer’s instructions.

### 2.4. Preparation of Cell-Laden Scaffolds 

The isolated hUC-MSCs with a concentration of ~10^5^ cells/mL were added to the hydrogel solution before cross-linking, as described above. Four different hydrogel concentrations from 0.6 % to 8% (*w*/*v*), notation by S1, S2, S3, and S4 were investigated. All scaffolds had the same volume and were seeded with an equivalent number of cells. The cell-laden scaffolds were then cultured in MSC NutriStem^®^ XF Medium (Biological Industries) at 37 °C in 5% CO_2_.

### 2.5. Viability Assessment of hUC-MSCs in the Scaffolds

The proliferation and development of cells in LunaGel scaffolds from day 1 to 3 after encapsulating cells into the scaffolds can be seen by microscopic observation. Additionally, the quantification of cells’ proliferation and activity were evaluated by the WST assay. The WST assessment is based on the amount of formazan dye formed proportional to the dehydrogenase activity with NADH and the number of active cells. Therefore, changes in cell viability can easily be detected spectroscopically using a plate reader. Specifically, the cell-hydrogel mixture was transferred to the 96-well microplate and cross-linked. Afterward, culture media was added, and the samples were cultured for 18, 24, 42, and 48 h. At the mentioned time points, 10 µL WST solution (Cell Counting kit-8, Dojindo, Kumamoto, Kyushu, Japan) was added to the samples. The sample OD was then measured by a Versa Max microplate reader at 450 nm. The WST assay was replicated 10 times with three technical replicates.

### 2.6. Surface Maker Analysis of the Cells in the Scaffold In Vitro

After five days of culture, cells of the S1, S2, S3, and S4 groups were obtained by enzymatically dissolving the LunaGel scaffold using an enzyme mix solution supplied by the manufacturer. The dissolved mixture was diluted with 5 mL of PBS and centrifuged at 2500 rpm for 4 min. Cell pellets were collected and stained with antibodies from the MSC analysis kit (BD Bioscience). The cell surface markers in the S1, S2, S3, and S4 groups were then analyzed by flow cytometry.

### 2.7. Sterility Assessment

The cell-free and cell-laden scaffolds were sent to the Institute of Drug Quality Control (Ho Chi Minh City, Vietnam) to test for sterility.

### 2.8. Assessment of Cell-Laden Scaffold Surface by SEM

The scaffolds were freeze-fractured and processed for SEM imaging as described elsewhere [20,21] (Leica EM ACE600 and Leica EM VCT500 freeze-fracture machine). Samples were then imaged using Cryo-scanning electron microscopy (Cryo-SEM, Biotechnology Center of Ho Chi Minh City).

### 2.9. Assessment of Scaffold’s Degradation 

To assess the degradation of the scaffold, 1 cm × 1 cm scaffolds were immersed in FPS and 2 mL PBS at 37 °C. The mass of the scaffold before immersing in FPB and PBS is denoted as m_1_. After one day, five days, ten days, and twenty days, scaffolds were weighed (m_2_). The remaining mass percentage (%M) after immersion was calculated as [%M = (m_2_/m_1_) × 100]. The experiment was repeated at least three times. 

### 2.10. Toxicity Assessment of LunaGel Scaffold

#### 2.10.1. Toxicity Assessment through Exposure

Fibroblast cell culture was used to evaluate the toxicity. The secretions or degradation products of the material will diffuse into the culture medium, which may or may not affect the cells in the culture dish. If the material is not toxic, the cell remains in a normal state of attachment and proliferation. Latex rubber was used as a positive control, while the negative control was a cell culture only. 

In particular, the 3T3 cells were cultured at a density of 10^6^ cells/mL in cell culture dishes in Dulbecco’s Modified Eagle Medium/Nutrient Mixture F-12 (DMEM/F-12) culture medium (Sigma–Aldrich, St. Louis, MO, USA) supplemented with 10% fetal bovine serum (FBS) (Sigma–Aldrich) at 37 °C in 5% CO_2_. The samples were placed in the center of the well that was covered with fibroblasts and incubated for 24 h in the cell culture cabinet. The samples’ size was equal to 1/10 of the well’s area. After one day, the cells’ death zone was digitally recorded under a light microscope (Nikon-TS2) at 0 h and 24 h.

#### 2.10.2. Toxicity Assessment through Scaffold Incubating Medium

The cell-free scaffold, cell-laden scaffold, and Latex rubber were incubated for 24 h in the cell culture medium. The medium was then collected to assess toxicity by the WST assay method, as described before [22]. The assay was repeated 12 times.

### 2.11. Animal Experimental Design

Twelve Balb/c mice (6 to 8 weeks old, weighing 27 to 32 g) were obtained from the Stem Cell Institute (Binh Duong province). All experiments were performed with ethical approval from the Animal Care and Use Committee at the Stem Cell Institute (Ref No.: 201201/SCI-AEC).

The cell-free and cell-laden scaffolds were prepared as mentioned above. The porcine tissues, used as a xenotransplantation comparison, were prepared from the pork obtained from the local market. Specifically, the pork was washed with saline solution and immersed in antibiotics, followed by sterilization with ethylene oxide gas. Then, the tissues were cut into sizes similar to the scaffold. The tissue samples were placed into a fresh plate covered with sterile saline solution before use.

Mice were subcutaneously transplanted with cell-free hydrogel scaffolds (n = 3), cell-laden scaffolds (n = 3), or porcine tissue (n = 3). The graft size was 1 cm × 1 cm × 5 mm. Anesthetized mice were shaved, and the material was transferred between the skin and abdominal muscle through a 1.5 cm incision. Gentamicin was applied to the wound. Three mice, without any surgery, were shaved at the corresponding area and used as the control. 

The mice were then monitored for changes in weight, appearance, and behavior. After 14 days, or when the mice’s conditions deteriorated, they were sacrificed to collect peripheral blood and the grafts for flow cytometry and histology analysis, respectively.

### 2.12. Flow Cytometry Analysis

Collected peripheral blood was stored in EDTA tubes. An amount of 100 µL of peripheral blood or bone marrow cell suspension was transferred into FACS tubes containing PharmingenStain Buffer (BSA), and cells were stained for 30 min with the following antibodies: CD4^+^ cells (FITC-conjugated ab269349 antibody); CD8^+^ cells (PE-conjugated ab25498 antibody); B cells (ab64088 for primary antibody and ab150077 for secondary antibody); natural killer (NK) cells (ab137059 for primary antibody and ab150077 for secondary antibody); neutrophils (FITC-conjugated ab53453 antibody), dendritic cells (Cat. No. 564882), and Monocytes/Macrophages (Mo/Ma) (ab33451 for primary antibody and ab150157 for secondary antibody). Red blood cells were lysed with Lysing Solution for 20 min at room temperature. Cells were washed and resuspended in FACS buffer and kept at 4 °C in the dark until flow cytometric analysis.

Stained cells were acquired on a BD FACSAria III flow cytometer (BD Biosciences), and flow cytometry data were analyzed with BD FACSDiva version 8.0.1 (BD Biosciences).

### 2.13. Masson’s Trichrome Staining

The skins above the graft were shaved before collecting the panniculus carnosus muscle-graft-skin complex and fixed in paraformaldehyde. Fixed samples were dehydrated in serial dilutions of ethanol before being embedded in paraffin wax. The 3 µm sections were used for trichrome staining. After rehydration, the slides were immersed in Bouin’s Fluid for 60 min and washed with distilled water. Then, the slides were stained with Weigert’s Iron Hematoxylin for 5 min and washed for 2 min under running water. Bieber Scarlet/Fuchsin acid solution was applied to the slides for 15 min and washed with distilled water. The slides were then differentiated in phosphomolybdic/phosphotungstic acid until collagen no longer displayed a red coloring. Aniline Blue solution was applied to the slides for 5–10 min and washed with distilled water. Slides were then immersed in acetic acid solution (1% *v*/*v*) for 3–5 min. Finally, samples were dehydrated in ethanol and mounted using Permount. Images were digitally captured using light microscopy (Nikon-TS2).

### 2.14. Immunohistology 

The 3 µm sections were deparaffinized and submersed in distilled water twice for 5 min. Next, the endogenous peroxidase of samples was deoxidized with 3% H_2_O_2_ solution for 5 min and washed with Tris-buffered saline (TBS) for 5 min. The samples were blocked for nonspecific proteins with bovine serum albumin for 5 min and washed with TBS for 5 min. The slides were further incubated with anti-huCD44 primary antibody for 60 min and washed with TBS twice for 5 min. The sample was then incubated with a secondary antibody for 30 min and washed with TBS twice for 5 min. The samples were coated with Diamino Benzidine solution for 10 min and washed under running water for 5 min. Afterward, the slides were stained with Hematoxylin for 5 s. Finally, the samples were dehydrated and covered. Images were digitally captured via light microscopy (Nikon-TS2).

### 2.15. Statistical Analysis

The data were processed by GraphPad Prism software version 9.0 (GraphPad Software, San Diego, CA, USA). The viability assessment of hUC-MSCs in the scaffolds via WST assay was analyzed by applying nested one-way ANOVA at each time point, followed by Turkey’s multiple comparisons test for differences between groups. The mice leukocytes were analyzed by applying one-way ANOVA, followed by Dunnett’s multiple comparisons test for differences between groups.

## 3. Results

### 3.1. Human Umbilical Cord-Derived Mesenchymal Stem Cells Isolation and Culture 

**Figure 1:**
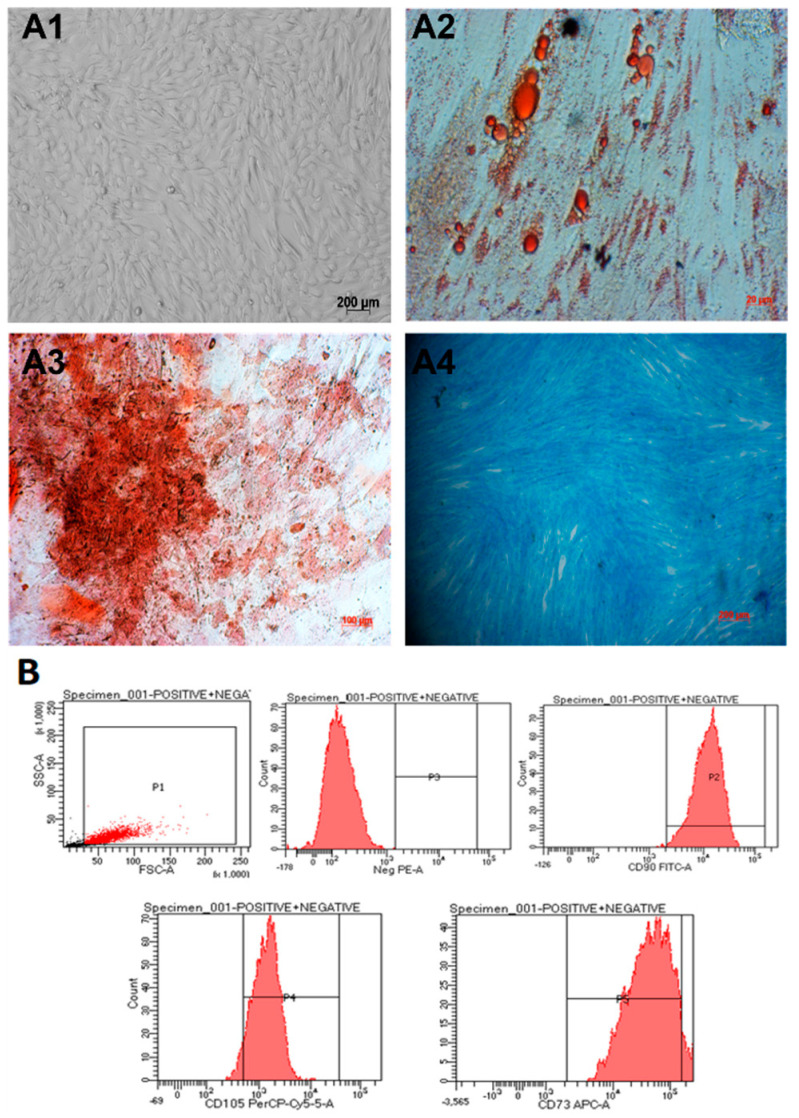
hUC-MSCs characterization. (**A1**) The cells exhibit a spindle shape, similar to fibroblasts. (**A2**–**A4**) After 21 days, the hUC-MSCs were able to differentiate into adipocytes, osteocytes, and chondrocytes, respectively. (**B**) The candidate cells are positive for CD105, CD73, and CD90 and negative for CD45, CD34, CD11b, CD19, and HLA-DR markers (negative cocktail).

Umbilical cord-derived mesenchymal stem cells (hUC-MSCs) were isolated from umbilical cord tissue and cultured as described before [19]. Cells were observed to spread evenly and exhibit fibroblast-like elongated and thin shapes (Figure 1(A1)). The cells were able to be differentiated into adipocytes (Figure 1(A2)), osteocytes (Figure 1(A3)), and chondrocytes (Figure 1(A4)). In addition, they expressed mesenchymal stem cell markers when analyzed using flow cytometry (Figure 1B). In particular, they were positive for CD105 (93.3%), CD73 (94.2%), and CD90 (99%) and negative for CD45, CD34, CD11b, CD19, and HLA-DR markers (Figure 1B). Thus, we concluded that we had obtained umbilical cord-derived mesenchymal stem cells.

Umbilical cord-derived mesenchymal stem cells were examined for Mycoplasma infection with MycoAlertTM Plus Mycoplasma Detection kit (Lonza). Negative results showed that hUC-MSC cells were not infected with mycoplasma (Supplementary Figure S1).

The doubling time for the isolated hUC-MSCs was calculated from the growth curve to be approximately 18 h (Supplementary Figures S2–S4).

### 3.2. Cell Encapsulation and Characterization

#### 3.2.1. Scaffold Preparation and Cell Encapsulation

LunaGel^TM^ is a photo-crosslinkable ECM that has been extensively used for growing cells in 3-dimensional scaffolds/hydrogels. The hydrogels were prepared according to the manufacturer’s instructions and involved mixing sterile LunaGel^TM^ ECM and photo-initiator in PBS and poured into a mold. A 405nm-light source (Luna Crosslinker device) was used to cross-link the hydrogels. The photo-crosslinking is relatively fast (1–10 min), and the crosslinked hydrogels were transparent and assumed the shape of the mold. Thus, this method has the potential to scale up for creating large hydrogels for the ultimate application in humans.

To create cell-laden scaffolds, hUC-MSCs were mixed into the hydrogel at different concentrations before photo-crosslinking.

#### 3.2.2. Assessment of the Viability and Proliferation of Cells within the Scaffold

**Figure 2:**
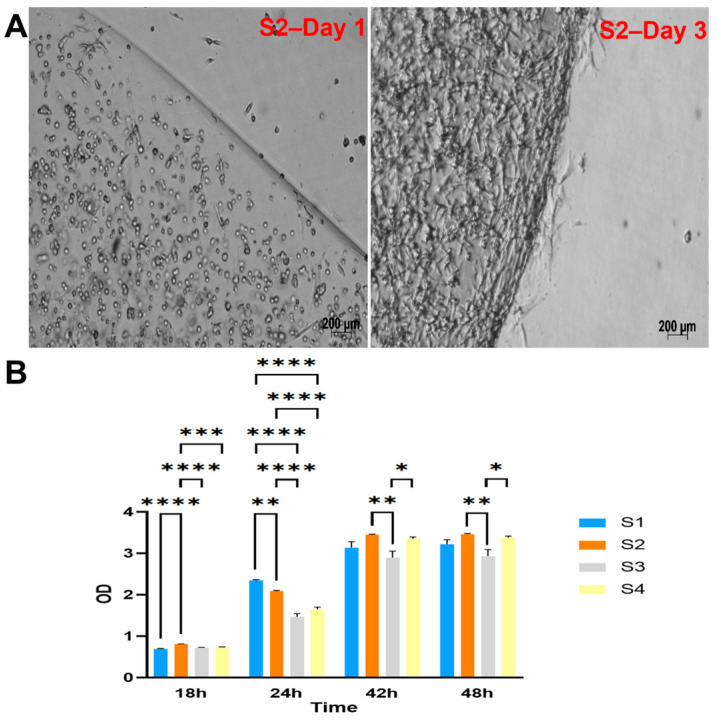
Cell viability and proliferation within LunaGel scaffolds using various methods. (**A**) via microscopic images. After being cross-linked, the created cell-laden scaffolds were placed in a 12-well plate individually and monitored under the light microscope for cell proliferation; and (**B**) by WST assay. For the WST assay, the cell–hydrogel mixture was added to the 96-well plate and cross-linked. The samples were cultured within the same plate, and WST reagents were added at 18, 24, 42, and 48 h. Sample OD was then measured at 450 nm. *: *p* ≤ 0.05; **: *p* ≤ 0.01; ***: *p* ≤ 0.001; ****: *p* ≤ 0.0001.

The four groups of cell-laden hydrogels S1, S2, S3, and S4 were monitored and observed under a microscope after one day and three days of culture. The cells appeared to proliferate within the scaffold well, as shown in Figure 2A and Supplementary Figure S6. 

The viability and proliferation of cells in the scaffolds were further assessed by WST assay (Figure 2B). The results showed the highest cell metabolism (indicated by the measured OD) in the S2 group after 2 days of culture.

#### 3.2.3. Characterization of Cells Inside the Scaffolds

**Figure 3:**
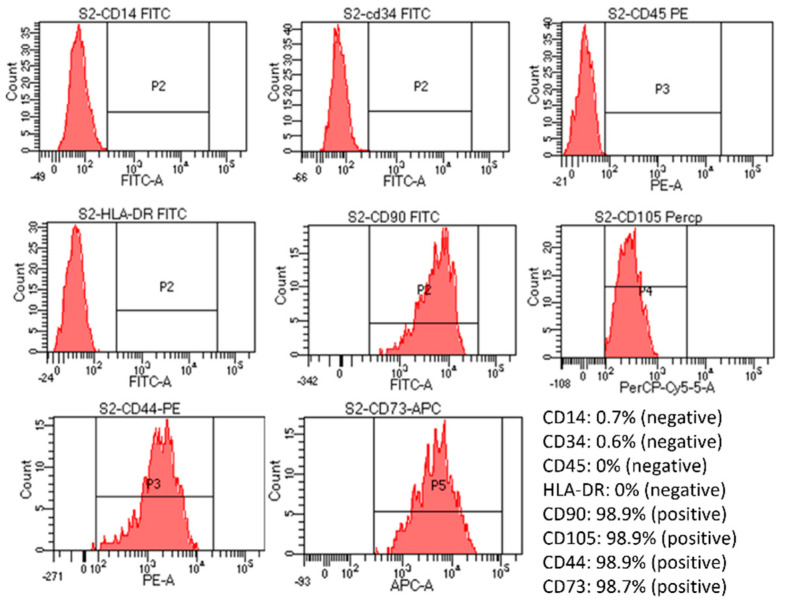
Evaluation of cell marker expression by flow cytometry of post-seeded hUC-MSCs in S2 sample. The created cell-laden scaffolds were cultured in MSC NutriStem^®^ XF Medium at 37 °C in 5% CO_2_. After being seeded into the scaffold for five days, the cell-laden scaffolds were dissolved by an enzyme mix solution supplied by the manufacturer. The dissolved mixture was diluted with 5 mL of PBS and centrifuged at 2500 rpm for 4 min. Cell pellets were collected and stained with antibodies from the MSC analysis kit. Results showed that the MSCs were still positive for CD90, CD105, CD44, and CD73 and negative for CD14, CD34, CD5, and HLA-DR.

Flow cytometry assessment of markers expression showed that the cells were positive for CD105, CD73, CD90, and CD44 (>90%) and negative for CD14, CD34, CD45, and HLA-DR markers (Figure 3). However, the positive percentage of cells from the S2 group was the finest, close to the control, and had the most negligible variation compared to cells in the other three groups (Supplementary Figure S7). Specifically, positive percentages of CD14, CD34, and CD45 were increased in the S1 sample; positive percentages of CD105 were reduced in the S3 sample; also, a high increase in CD34%, an increase in CD45, and a slight decrease in CD105 were noticed in S4 sample. Thus, when combined with the viability and proliferation assessment results, the S2 sample gave the best results out of the four samples.

#### 3.2.4. Sterility Assessment

The LunaGel scaffolds and stem cell sheets passed the sterility test according to the Vietnamese Pharmacopeia V standard (Supplement Figure S8).

#### 3.2.5. Cell Encapsulated Scaffold Surface Assessment Using Scanning Electron Microscopy (SEM)

**Figure 4:**
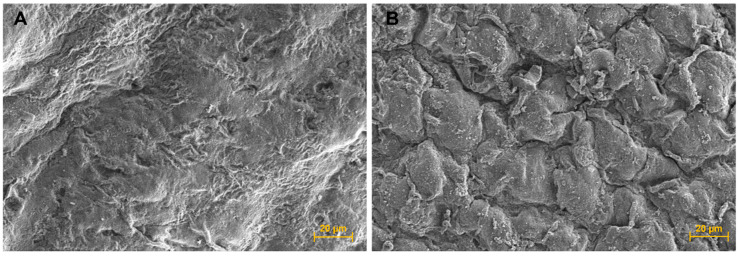
Scanning electron microscope (SEM) micrographs. (**A**) Scaffold surface; 600× magnification, and (**B**) Cell sheet surface; 600× magnification.

The SEM micrograph of the cell-laden scaffolds showed a different morphology to the cell-free scaffold surface and appeared to show that some encapsulated hUC-MSC cells were closed to the scaffold surface (Figure 4B). 

#### 3.2.6. Degradability of the Scaffolds

After 0, 5, 10, and 20 days of being submersed in FBS or PBS, the scaffolds remained stable, and their weight appeared to have slightly increased, indicating that the scaffolds were not degraded (Supplement Figure S10).

#### 3.2.7. Toxicity Assessment

As photo-initiators were used in the preparation of scaffolds, we conducted toxicity evaluation of the cell-laden scaffolds as these could potentially be used in applications such as sub-cutaneous implantation for reconstructive surgeries. The cell-free scaffolds and cell-laden scaffolds were assessed for toxicity according to ISO-10993-5, including toxicity assessment through exposure and toxicity assessment through the medium extract.

##### Toxicity Assessment through Exposure

**Figure 5:**
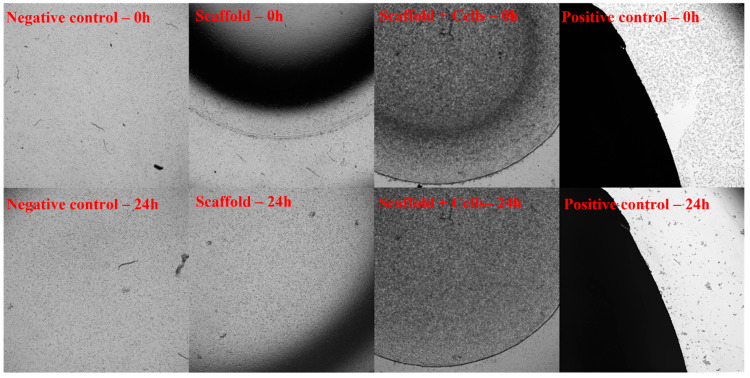
Toxicity assessment through exposure according to ISO-10993-5. The 3T3 cells were cultured in cell culture dishes at 37 °C in 5% CO_2_. The cell-free scaffold (Scaffold), cell-laden scaffold (Scaffold + Cells), and Latex rubber (Positive control) were placed in the center of a well that was covered with fibroblasts and incubated for 24 h. The sample’s dimension is equal to 1/10 of the well’s area. The negative control was a cell culture only. The 3T3 proliferation was recorded under the microscope at 0 h and 24 h. At 0 h, when the material had contacted with 3T3 cells, all 4 samples (Negative control, Positive control, Scaffold, and Scaffold + Cells) showed that the cells were still covering the culture dishes. After 24 h of exposure, there was a significant difference between the positive control and the remaining 3 samples. While the cells in the positive control sample almost died out, cells in other groups still proliferated and developed normally. The results showed that both the cell-free and cell-laden scaffolds were not toxic when exposed to the cells.

After 24 h of exposing fibroblast cell layers on tissue culture plates to the cell-free scaffolds and cell-laden scaffolds, no signs of toxicity were observed (Figure 5).

##### Toxicity Assessment through Scaffold Incubating Medium Extract

The cell-free scaffolds or cell-laden scaffolds culture medium were collected and added to the fibroblast cell cultures. After 24 h, the extract’s toxicity was assessed by WST assay. The results showed that test cells grown in either cell-free or cell-laden scaffolds showed a viability of over 70% (Supplementary Table S5). 

After assessing the toxicity through exposure and incubating medium extract according to ISO-10993-5, the Luna scaffolds and cell sheets met the non-toxic standards.

### 3.3. Subcutaneous Implantation of Cell-Free and Cell-Laden Scaffolds

**Figure 6:**
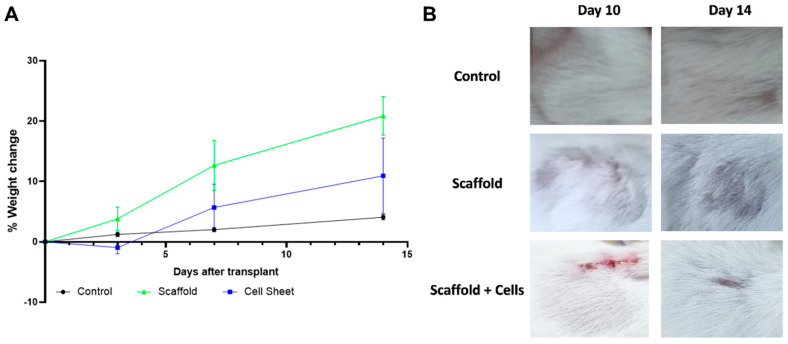
Mice condition monitoring post-implantation. (**A**) % weight change of mice engrafted with scaffold, cell-laden scaffold, and control mice. (**B**) The area above the implant site of mice engrafted with scaffold and cell-laden scaffold. Balb/C mice were implanted with the cell-free scaffold (n = 3) and cell-laden scaffold (n = 3). Three mice were shaved at the corresponding implanting area and were used as the control. The mice were weighed at days 0, 3, 7, and 14 after the implantation for any sign of severe weight loss. The fur regeneration rate was also monitored. Results showed that the mice gained weight normally, and the fur was fully regenerated after 14 days.

Mice implanted with cell-free scaffolds and cell-laden scaffolds remained stable with normal activity and weight gain (Figure 6A). The implanted materials appeared not to affect the upper skin area, as the fur fully regenerated within 14 days (Figure 6B). 

**Figure 7:**
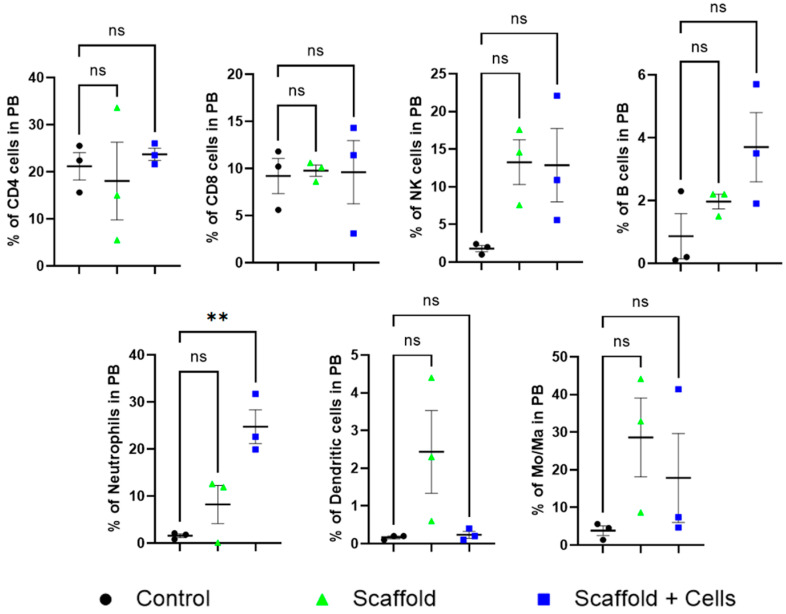
Peripheral blood leukocytes analysis. ns: nonsignificant (*p* > 0.05); **: *p* ≤ 0.01. After 14 days of implantation, the mice’s peripheral blood was collected for leukocyte analysis. The mice CD4+, CD8+, NKs, B cells, neutrophils, DCs, and Mo/Ma were measured by flow cytometry using specific antibodies, as mentioned in Section 2.12.

Peripheral blood analysis did not find any statistical differences in the level of CD4+ cells, CD8+ cells, B cells, NK cells, dendritic cells, and monocytes/macrophages among the mice. However, the neutrophils cell-laden scaffold group was noticeably higher (Figure 7). 

### 3.4. Histology

**Figure 8:**
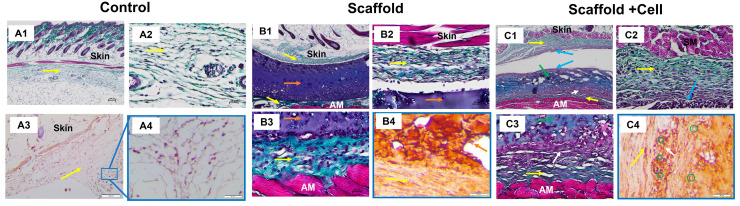
Histology staining. (**A**) Control group: (**A1**) Trichrome staining at 4× magnification, 100 µm scale; (**A2**) Trichrome staining at 20× magnification, 25 µm scale; (**A3**) Immunohistochemistry staining against hu-CD44 at 10× magnification, 100 µm scale; and (**A4**) Immunohistochemistry staining against hu-CD44 at 20× magnification, 50 µm scale. (**B**) Mice implanted with LunaGel scaffold: (**B1**) Trichrome staining at 4× magnification, 100 µm scale; (**B2**,**B3**) Trichrome staining at 20× magnification, 25 µm scale; and (**B4**) Immunohistochemistry staining against hu-CD44 at 20× magnification, 50 µm scale. (**C**) Mice implanted with cell-laden scaffold: (**C1**) Trichrome staining at 4× magnification, 100 µm scale; (**C2**,**C3**) Trichrome staining at 20× magnification, 25 µm scale; and (**C4**) Immunohistochemistry staining against hu-CD44 at 20× magnification, 50 µm scale. Yellow arrow, collagen layer; orange arrow, LunaGel scaffold; green arrow, cell-laden scaffold; blue arrow, cell concentration area on the upper surface of the cell sheet and adjacent regions; white arrow, cell sheet adhering to the collagen layer above the abdominal muscle with a concentration of cells; green circle, hu-CD44+ cells; AM, abdominal muscle.

Histology analysis showed that the implanted materials were surrounded by collagen fibers (Figure 8(B1,C1)), while in the porcine tissue group, they were massively infiltrated with leukocytes (Supplementary Figure S13). The cell-laden scaffolds seemed to attach to the collagen layer above the abdominal muscle in the presents of various cells (Figure 8(C3)). In addition, there was a concentration of cells on the cell-laden scaffold’s upper surface and the bordering tissues (Figure 8(C1)). 

Immunohistochemistry analysis showed positive for the hu-CD44 marker in the cell-laden scaffold group, indicating that the transplanted cells survived after 14 days. The hu-CD44^+^ MSCs were still identified within the cell-laden scaffolds. Importantly, we found that the MSCs were able to migrate to the adjacent tissues as the hu-CD44^+^ cells were also found in the collagen layer surrounding the cell-laden scaffolds (Figure 8(C4)).

## 4. Discussion

In recent years, mesenchymal stem cells have been one of the brightest candidates in the field of regenerative medicine for the treatment of damaged or diseased tissues in the skin, bones, cartilage, and cardiovascular system [23,24,25]. In addition to the capability to differentiate into many different cell lines, which helps diversify the treatment for diseases, mesenchymal stem cells also have the ability to modulate the host’s immune response and, thus, could minimize the risk of rejection [26].

Mesenchymal stem cells are found in various body parts, such as bone marrow, adipose tissue, and umbilical cord. In this study, we used umbilical cord-derived mesenchymal stem cells because they are abundantly available as the umbilical cord is usually discarded after the baby is born and because of the easy, non-invasive, and ethically accepted collection method [27]. In addition, being of neonatal origin, hUC-MSCs exhibit a longer lifespan, higher proliferation rates, and greater differentiation potential compared to adult tissue-derived MSCs [28].

In our study, the results showed that our method of isolation was successful in obtaining umbilical cord mesenchymal stem cells that (1) exhibited a spindle, fibroblast-like shape (Figure 1(A1)), (2) had the surface markers positive for CD105 (93.3%), CD73 (94.2%), CD90 (99%), and negative for CD45, CD34, CD11b, CD19, and HLA-DR markers (Figure 1B); (3) exhibited the ability to be differentiated into many cell lines, such as adipocytes, osteocytes, and chondrocytes (Figure 1(A2–A4)). The mesenchymal cells were also tested for mycoplasma before further experimentation (Supplementary Figure S1).

A key factor in many stem cell-based regenerative medicine applications is the scaffold to encapsulate the cells. The scaffold must support cellular adhesion and proliferation and be conducive to cell differentiation. We chose the photo-crosslinkable extracellular matrix (LunaGel™) as the scaffold material because of those reasons and because it can be photo-crosslinked to give the desired porosity and stiffness.

The viability of the seeded cells showed that the scaffold had created a suitable environment and structure for cell proliferation (Figure 2). Flow cytometry analysis of surface markers post-seeding once again confirmed that the cells within the scaffold were mesenchymal stem cells as they showed positive for CD105, CD73, CD90, and CD44 markers (>90%) and negative for CD14, CD34, CD45, and HLA-DR markers (Figure 3), thus, showing that proliferated cells within the scaffold retained their stem cell-ness.

In vivo, after the sub-cutaneous implantation of the cell-free and cell-laden scaffolds, all mice showed normal behavior and weight gain (Figure 6A). Histology analysis showed more details of the host response to the implanted cell-free scaffolds and cell-laden scaffolds. The neutrophil level in the cell-laden scaffold group was significantly higher (Figure 7). It has been pointed out that MSCs can increase the viability of neutrophils by rescuing them from apoptosis fate; however, the neutrophils’ infiltration was also reduced and suppressed by various MSCs’ cytokines [29]. Moreover, the neutrophils also secrete growth factors and chemokines (lipoxin, resolvin) that degrade and reduce inflammation [30]. In addition, metalloproteinase 9 (MMP-9) secreted by neutrophils has also been shown to have the ability to restructure the extracellular matrix around the premise material to promote vascular proliferation and regeneration, tissue formation, wound healing, and anti-fibrosis [31,32].

After 14 days in vivo, the scaffold structure remained visible, and thin layers of collagenous fibers were found surrounding the grafts (Figure 8(B1,C1)). This is expected as nearly all engrafted materials will exhibit foreign body reaction (FBR) in vivo, which consists of a fibrous capsule in combination with persistent mild inflammation [33,34,35]. Significantly, Swartzlander and colleagues have also observed that the fibrous capsule surrounding their acellular scaffolds was visibly denser than in those seeded with MSCs, thus, pointing out the fact that MSCs could modulate host’s immune response and reduce fibrotic formation in FBR. The collagen surrounding the cell sheet could also be due to the growth factors secreted by the hUC-MSCs that were known to improve fibroblast migration and survival, as well as fibroblast ECM deposition [36]. The TGF-β3 cytokine released by MSCs is thought to have effects on fibroblast proliferation, collagen accumulation, ECM regulation, and antifibrotic [37]. Derived products of MSCs, such as exosomes, also facilitated the proliferation and migration of fibroblasts [38].

Noticeably, a connective tissue layer was found to form between the material and the surrounding collagen layers in the cell-loaded scaffold group (Figure 8(C2,C3)), which was not observed in the cell-free scaffold group. We postulated that this region of connective tissue was derived from the implanted MSCs. Although mesenchymal stem cells have been demonstrated for their immunomodulation ability [39], they might not be able to bypass the species–species barrier. It has been demonstrated that transplanted adult human mesenchymal stem cells were rejected and could not be found after one week of being engrafted in Sprague-Dawley rats and associated with massive macrophage infiltration [40]. Accordingly, immunohistochemistry staining with human anti-CD44 antibodies showed the presence of CD44-positive MSCs inside the newly formed connective tissue and material (Figure 8(C4)). This result supports our hypothesis about the survival of MSCs in the scaffolds after implantation and their migration to surrounding tissues. We, thus, suggested that the scaffold had protective effects on the encapsulated cells, protecting them from the host immune system [41].

Although the performance of the cell-laden scaffolds in vivo needs to be investigated for longer engraftment duration, the foreign body capsule appeared thin, and the reaction site was relatively quiescent, strongly suggesting the potential for tissue regeneration [34].

## 5. Conclusions

In summary, we have developed a simple method to encapsulate and deliver hUC-MSCs for applications in regenerative medicine. The photo-crosslinkable ECM (LunaGel) can readily be mixed with hUC-MSCs and processed into cell-laden scaffolds for cellular expansion and implantation. When implanted subcutaneously in mice, the cell-laden scaffolds were found to cause mild inflammation reaction, and the cells were found to survive and migrate to the surroundings and form new tissue. Thus, this method could be promising for tissue engineering applications in areas such as reconstructive surgeries.

## Data Availability

The data presented in this study are available on request from the corresponding author.

## Supplementary Materials

The following supporting information can be downloaded at: https://www.mdpi.com/article/10.3390/biomedicines11041158/s1, Figure S1: Mycoplasma detection; Figure S2: The growth curve of the hUC-MSC cell line that was investigated by automatic cell counting method using the Vi-Cell system; Table S1: The growth curve assessment data of the hUC-MSC cell line that was investigated by automatic cell counting method using the Vi-Cell system; Figure S3: The growth curve of the hUC-MSC cell line in 30 h that was investigated by the trypan blue staining method; Table S2: The growth curve assessment data of the hUC-MSC cell line investigated by trypan blue staining method; Figure S4: The growth curve of the hUC-MSC cell line in 30 h using INCell Analyzer 2500 system; Table S3: Growth curve assessment data of the hUC-MSC cell line that was investigated by visual monitoring method using the INCell Analyzer 2500 system; Figure S5: The produced LunaGel scaffolds (A) and Scaffold + Cells (B); Figure S6: Microscopic images of cell viability and proliferation within LunaGel scaffolds; Table S4: WST assay results for evaluating cell viability and proliferation on the scaffold; Figure S7: Evaluation of cell markers expression by flow cytometry of post-seeded hUC-MSCs; Table S5: Toxicity assessment through the medium extract; Figure S8: The certificate of the LunaGel scaffold (A) and cell-laden scaffold (B) passed the sterility test according to the Vietnamese Pharmacopeia V standard; Figure S9: Before and after drying of LunaGel scaffolds that were soaked in FBS and PBS for 20 days; Figure S10: LunaGel scaffold degradability in FBS and PBS; Table S6: Data on the evaluation of the degradability of LunaGel scaffold in FBS and PBS; Figure S11: (A) % weight change of mice engrafted with porcine tissue. (B) The area above the implant site of mice engrafted with porcine tissue; Figure S12: Peripheral blood leukocytes analysis of porcine tissue implant group; Figure S13: Histology staining of mice implanted with porcine tissue.
