# Supplementary material for: Encapsulation of Human Umbilical Cord Mesenchymal Stem Cells in LunaGel Photocrosslinkable Extracellular Matrix and Subcutaneous Transplantation in Mice
Source: Biomedicines. 2023 Apr 12;11(4):1158. doi: 10.3390/biomedicines11041158 (PMC10135450; doi:10.3390/biomedicines11041158)
Supplement: Supplementary file 1 [file biomedicines-11-01158-s001.zip › biomedicines-2277145-supplementary.pdf]

Supplementary Figure

Protocol:	MycoAlertPLUS			
Sample	Read A [RLU)	Read B [RLU)	Ratio [RLU/s] Read B/Read A	Result
Positive	4	14	3.5	CONTAMINATED
Negative	5	1	0.2	CLEAN
S1	132	34	0.3	CLEAN
S2	115	35	0.3	CLEAN
S3	123	33	0.3	CLEAN

Supplementary Figure S1. Mycoplasma detection. The hUC-MSCs were not infected with mycoplasma when tested by the MycoAlertTM Plus Mycoplasma Detection kit. The test could detect up to 200 mycoplasma species. Viable mycoplasma in a test sample is lysed, and the released mycoplasmal enzymes react with the MycoAlert® PLUS Substrate, catalyzing the conversion of ADP to ATP. By measuring the level of ATP in a sample both before (read A) and after the addition of the MycoAlert® PLUS Substrate (read B), if mycoplasma enzymes are not present, Read B shows no increase over Read A, if mycoplasma is present, Read B would increase.

Supplementary Figure

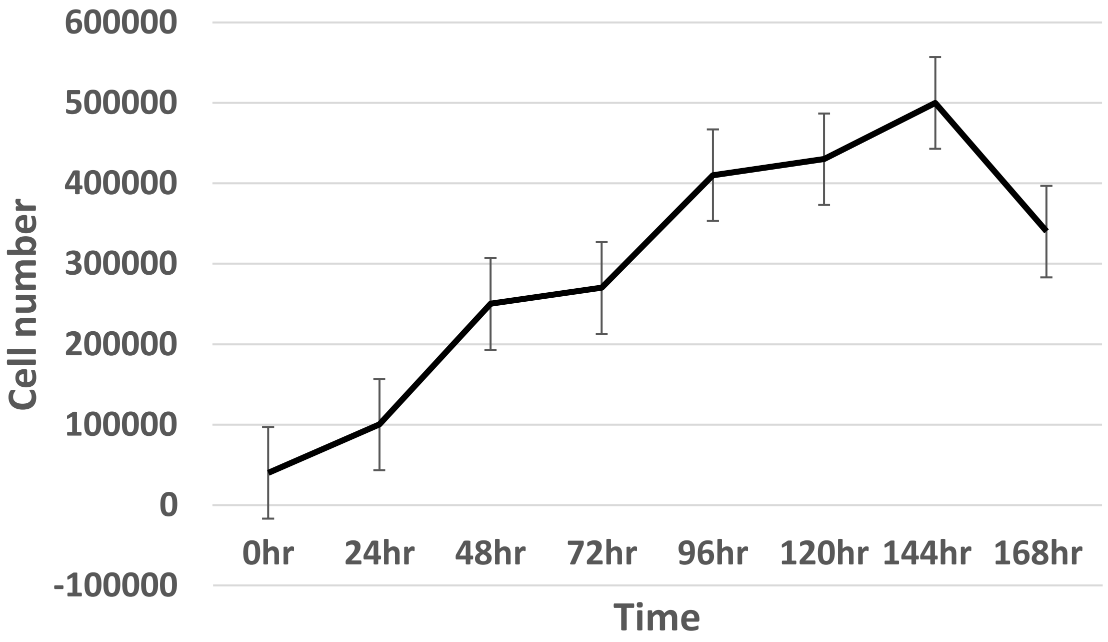

Supplementary Figure S2. The growth curve of the hUC-MSC cell line that was investigated by automatic cell counting method using theVi-Cell system. Doubling time (td) is calculated as:

N(t) = N0*2^(t/td)

N(24) = N0*2^(24/td)

100000 = 40000*2^(24/td)

Thus, **td = 18,155 hours**

Supplementary Table

Supplementary Table S1. The growth curve assessment data of the hUC-MSC cell line that was investigated by automatic cell counting method using the Vi-Cell system. This table corresponds to Supplementary Figure S2.

Time	Number of cells(x10^6)			Average number of cells
	Well 1	Well 2	Well 3	
**0hr**	0.10	0.1	0.089	96000
**24hr**	0.2	0.25	0.25	233000
**48hr**	0.45	0.27	0.34	353000
**72hr**	0.43	0.41	0.43	423000
**96hr**	0.28	0.43	0.36	357000
**120hr**	0.53	0.5	0.45	493000
**144hr**	0.4	0.34	0.45	397000

Supplementary Figure

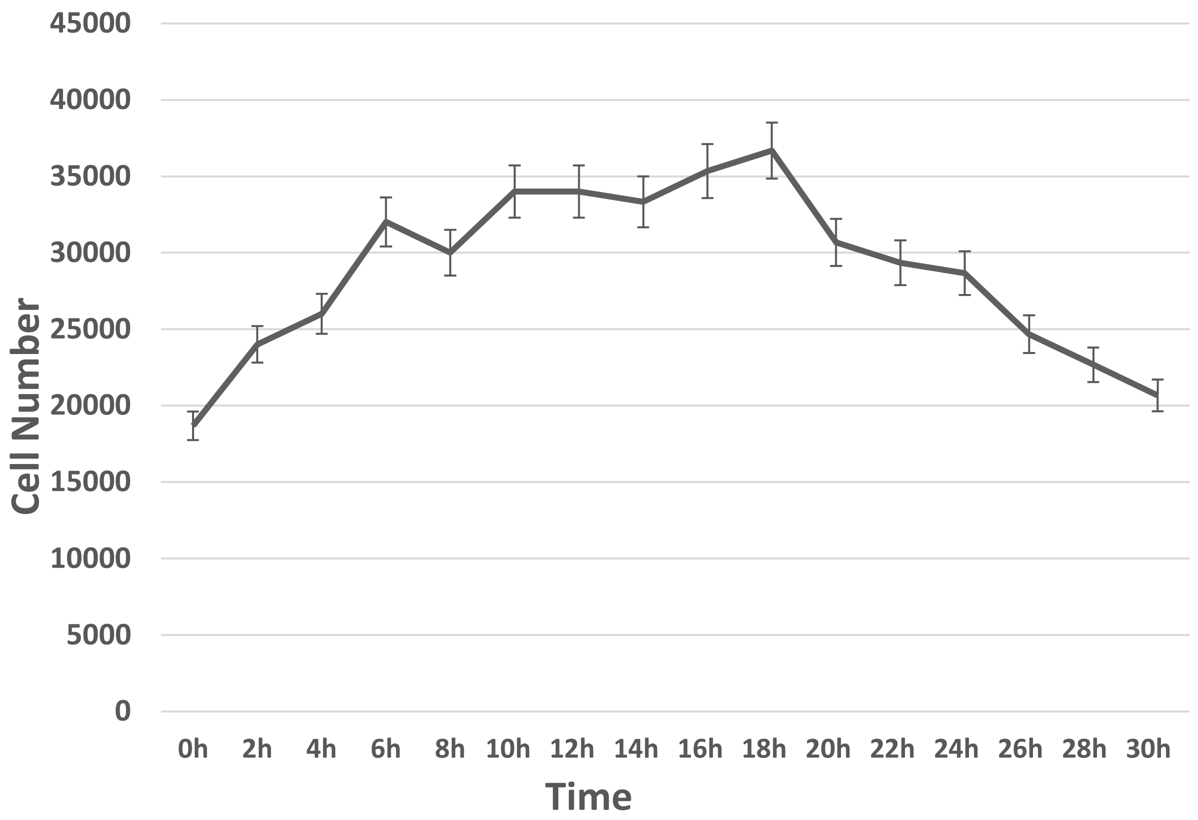

Supplementary Figure S3. The growth curve of the hUC-MSC cell line in 30 hours that was investigatedby the trypan blue staining method. Doubling time = 18*ln(2)/ln(36667/18667) = 18,5 hours.

Supplementary Table

Time	Well	Number of cells in counting chamber 1	Number of cells in counting chamber 2	Average number of cells
0h	1	4	3	
	2	6	6	
	3	4	5	18667
2h	1	6	6	
	2	6	6	
	3	5	7	24000
4h	1	6	7	
	2	7	7	
	3	5	7	26000
6h	1	7	7	
	2	7	9	
	3	8	10	32000
8h	1	8	7	
	2	7	7	
	3	7	9	30000
10h	1	7	9	
	2	8	8	
	3	9	10	34000
12h	1	8	10	
	2	8	9	
	3	7	9	34000
14h	1	7	9	
	2	8	8	
	3	9	9	33333
16h	1	8	8	
	2	8	10	
	3	10	9	35333
18h	1	10	9	
	2	8	10	
	3	9	9	36667
20h	1	8	7	
	2	6	8	
	3	10	7	30667
22h	1	7	8	
	2	8	7	
	3	8	6	29333
24h	1	6	7	
	2	9	7	
	3	6	8	28667
26h	1	5	5	
	2	6	8	
	3	6	7	24667
28h	1	6	6	
	2	5	7	
	3	4	6	22667
30h	1	5	6	
	2	5	7	
	3	4	4	20667

Supplementary Table S2. The growth curve assessment data of the hUC-MSC cell line investigated by trypan blue staining method. This table corresponds to Supplement Figure S3.

Supplementary Figure

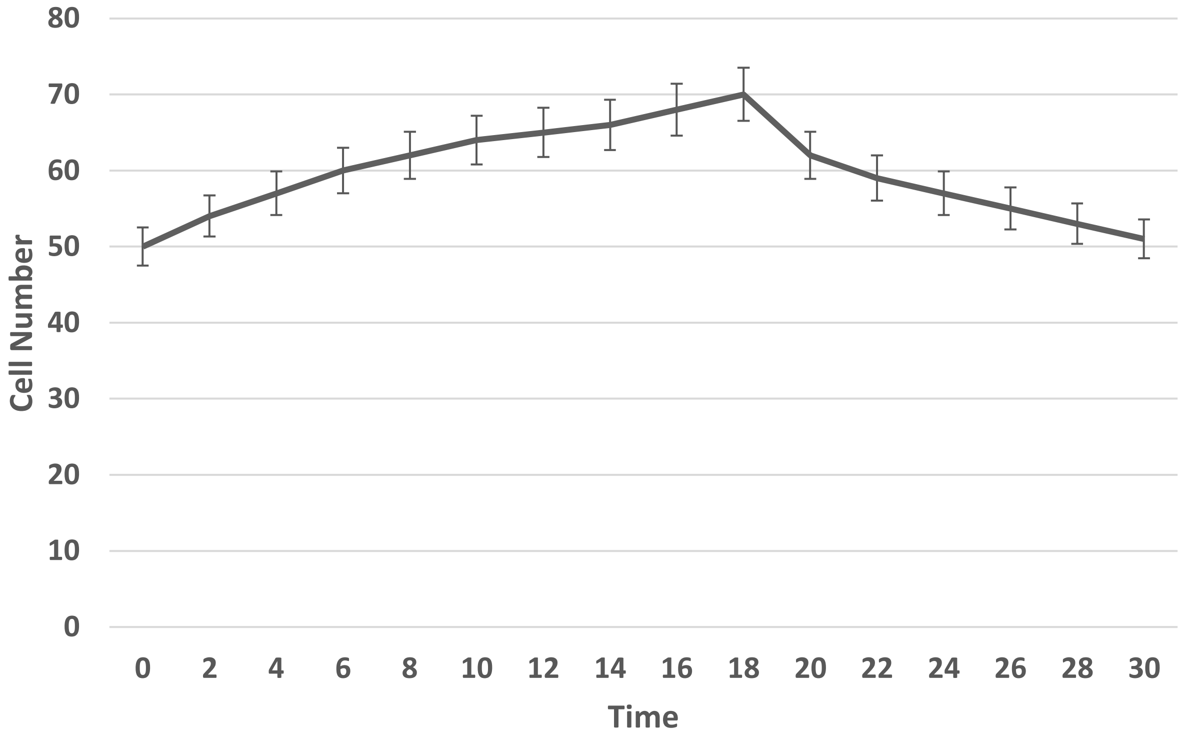

Supplementary Figure S 4. The growth curve of the hUC-MSC cell line in 30 hours using INCell Analyzer 2500 system. The cell duplication was visually recognised after **18 hours**.

Supplementary Table

Supplementary Table S 3. Growth curve assessment data of the hUC-MSC cell line that was investigated by visual monitoring method using the INCell Analyzer 2500 system. This table corresponds to Supplement Figure S4.

Time	Average number of cells in wells of C row	Average number of cells in wells of D row	Average number of cells in wells of E row	Average number of cells in wells of F row	Average number of cells
0h	51	53	48	48	50
2h	53	58	52	51	54
4h	56	62	55	53	57
6h	58	66	59	56	60
8h	60	69	60	59	62
10h	62	70	62	61	64
12h	63	72	62	61	65
14h	65	73	65	62	66
16h	67	74	65	64	68
18h	70	76	68	64	70
20h	61	67	60	59	62
22h	58	64	58	56	59
24h	56	62	56	55	57
26h	54	59	55	53	55
28h	51	55	53	52	53
30h	49	53	51	49	51

Supplementary Figure

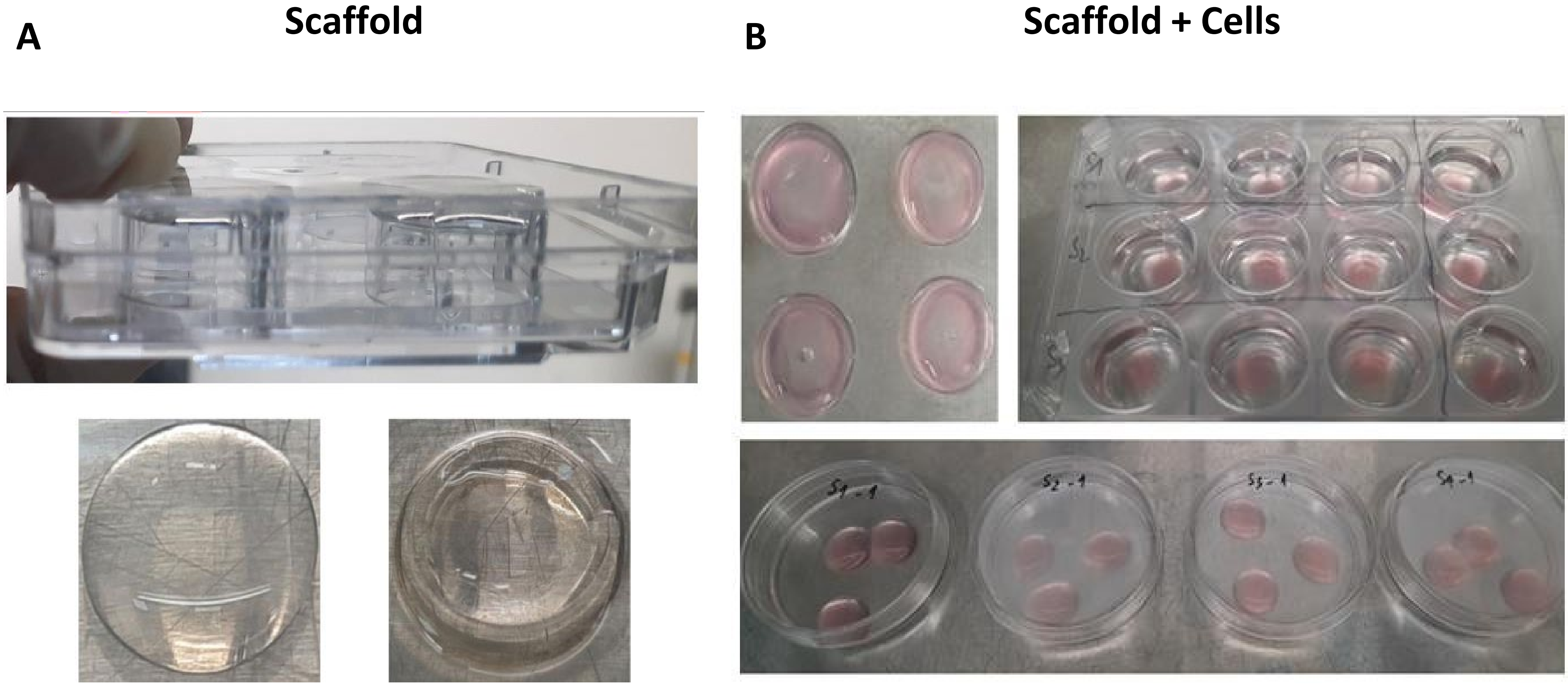

Supplementary Figure S5. The produced LunaGel scaffolds (A) and Scaffold + Cells (B).

Supplementary Figure

The cell-free scaffold and cell-laden scaffold assumed the shape of the mold.

Supplementary Figure

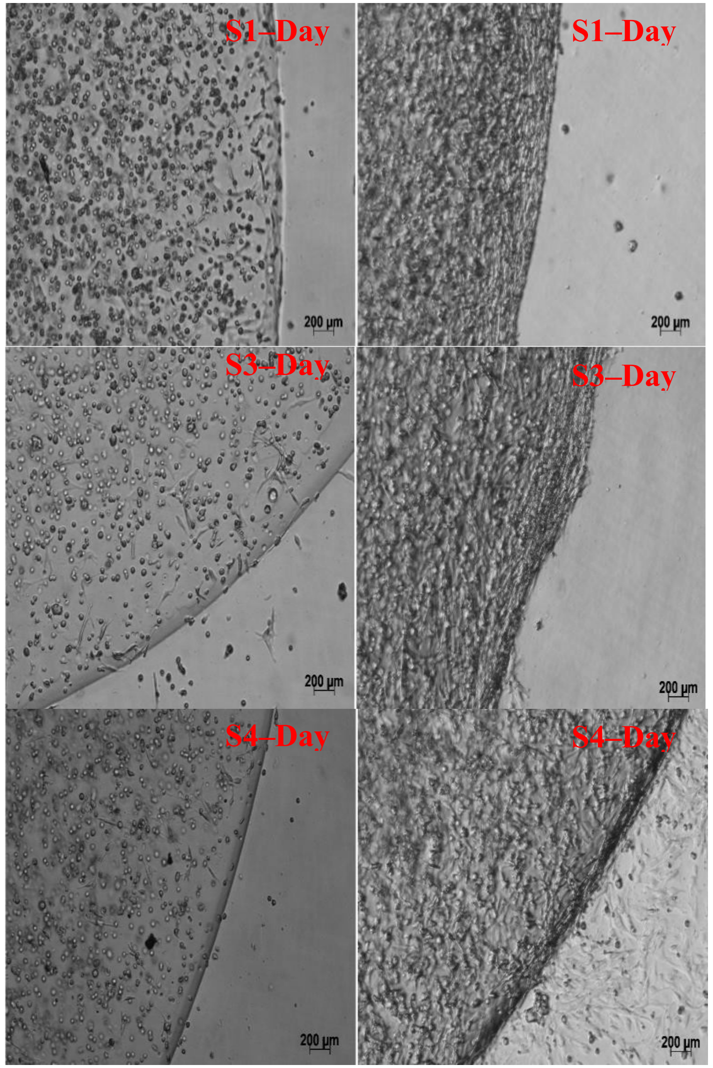

Supplementary Figure S 6. Microscopic images of cell viability and proliferation within LunaGel scaffolds. Microscopic observation showed that in all samples, the cells survived after linking and continued to spread and proliferate densely within the scaffold.

Supplementary Table

Supplementary Table S4. WST assay results for evaluating cell viability and proliferation on the scaffold. This table corresponds to Figure 2B. The results showed that the highest cell metabolism(indicated by the measured OD) in the S2 group after 2 days of culture.

Sample / Time	S1	S2	S3	S4
**18h**	0.69449	0.806887	0.721123	0.73319
**24h**	2.343507	2.08624	1.468617	1.649663
**42h**	3.136903	3.44686	2.893773	3.355423
**48h**	3.219607	3.462753	2.933607	3.38114

Supplementary Figure

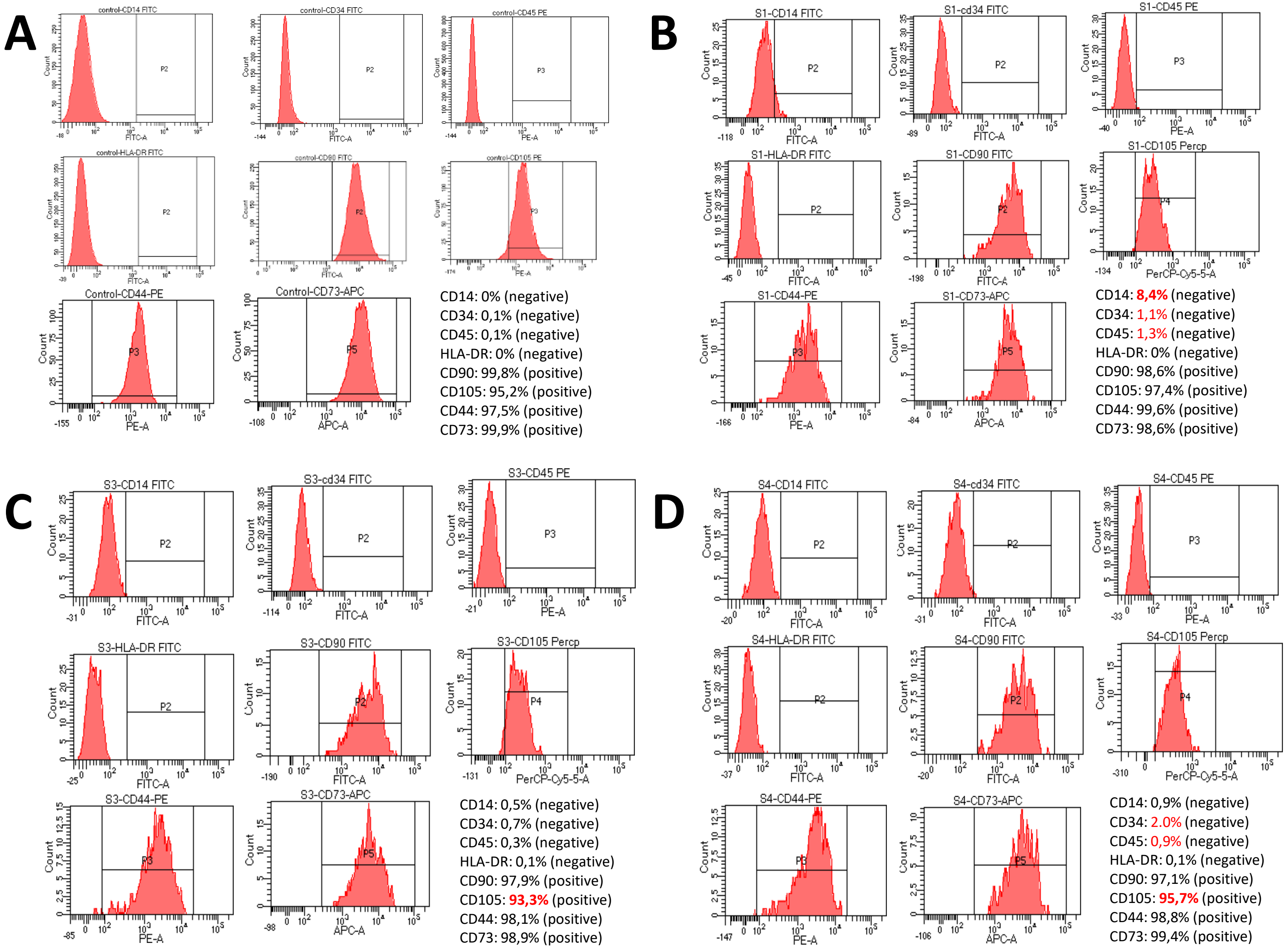

Supplementary Figure S7. Evaluation of cell markers expression by flow cytometry of post-seeded hUC-MSCs. After being seeded into the scaffold for five days, the MSCs still positive for CD90, CD105, CD44, and CD73 and negative for CD14, CD34, CD5, and HLA-DR. (A) Unseeded hUC-MSCs. (B) hUC-MSCs seeded in the S1 sample. (C) hUC-MSCsseeded in the S3 sample. (D) hUC-MSCs seeded in the S4 sample. The positive percentages of CD14, CD34, and CD45 were increased in the S1 sample; positive percentages of CD105 were reduced in the S3 sample; also, a high increase in CD34%, an increase in CD45, and a slight decrease in CD105 were noticed in S4 sample.

Supplementary Table

Supplementary Table S5. Toxicity assessment through the medium extract. Substances are capable of causing acute toxicity when % of living cells < 70%. The % Living cells = (OD sample / OD negative control) x 100. Thus, the cell-free scaffold and cell-laden scaffold do not toxic to the cell.

	Negative control	Positive control	Scaffold	Cell sheet
OD value after 24 hours	3.677	2.379	3.109	3.283
% of viable cells after 24 hours	100%	64.7%	84.6%	89.3%

Supplementary Figure

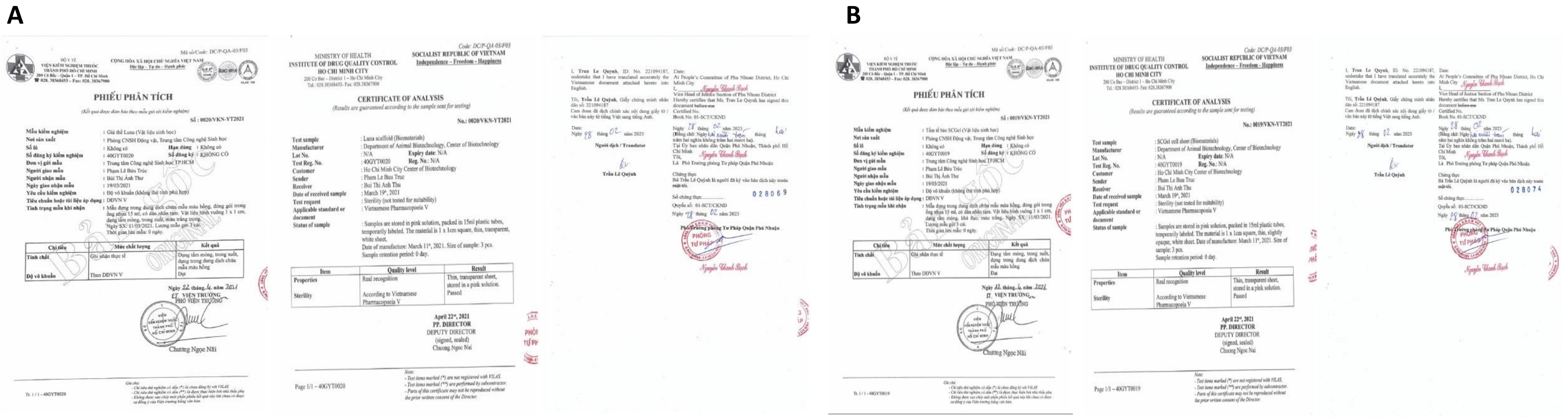

Supplementary Figure S8. The certificate of the LunaGel scaffold (A) and cell-laden scaffold (B) passed the sterility test according to the Vietnamese Pharmacopoeia V standard. The original documents were translatedinto English.

Supplementary Figure

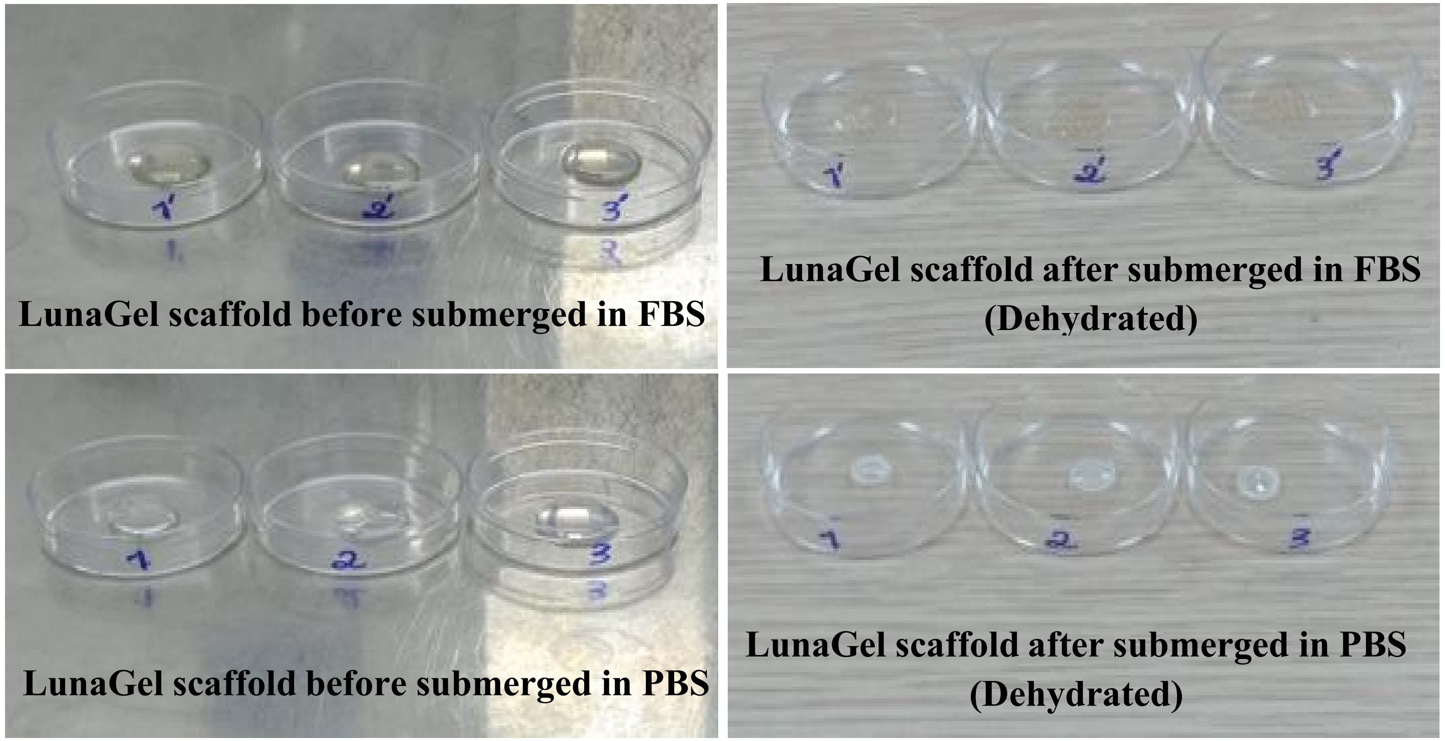

Supplementary Figure S9. Before and after drying of LunaGel scaffolds that were soaked in FBS and PBS for 20 days. The scaffolds were dried to identify the dry mass.

Supplementary Figure

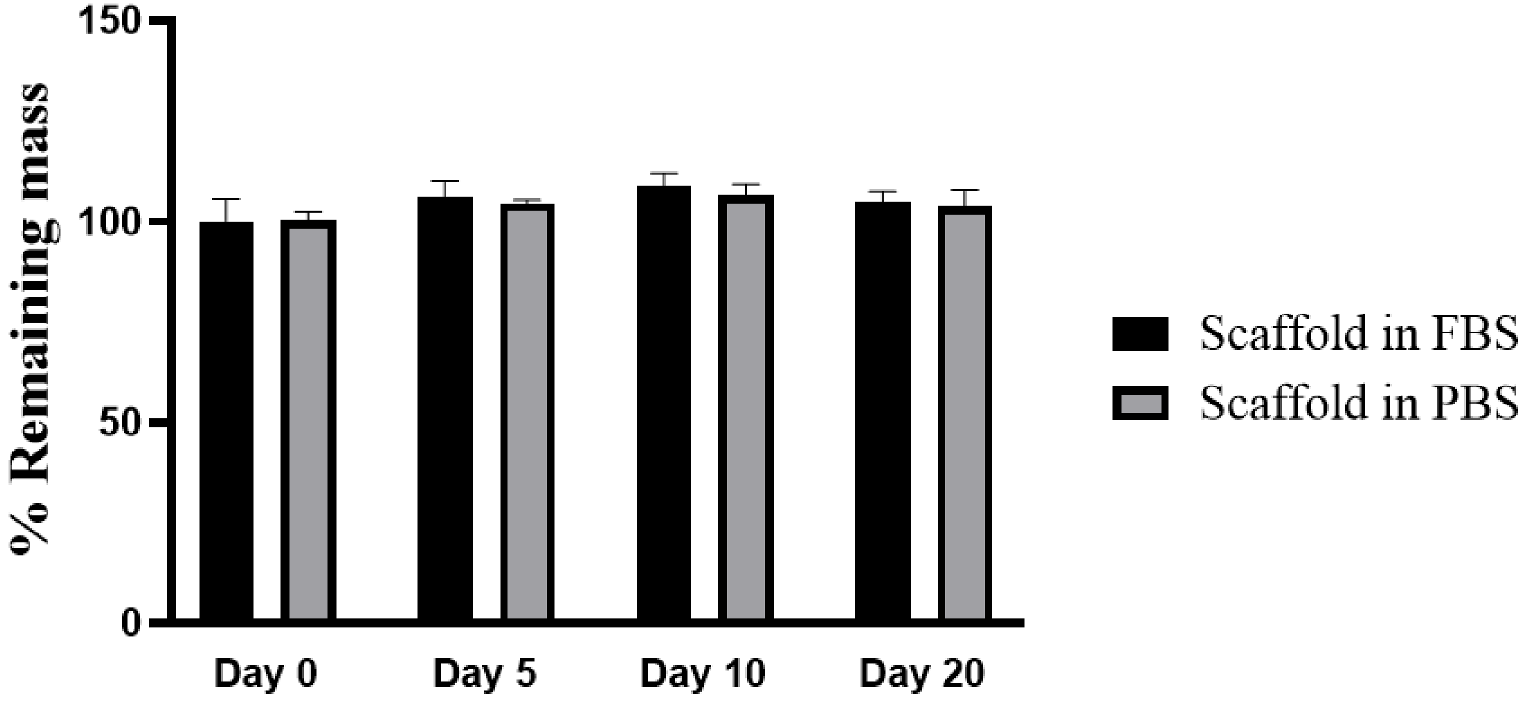

Supplementary Figure S10. LunaGel scaffold degradability in FBS and PBS. The degradability of the scaffold was assessed by measuring the dry mass of the scaffold before and after submerged in FBS and PBS.

Supplementary Table

Supplementary Table S6. Data on the evaluation of the degradability of LunaGel scaffold in FBS and PBS. **This table corresponds to Supplementary Figure S10.**

ASSESSMENT OF THE BIODEGRADABILITY OF THE LUNAGEL SCAFFOLD						
**Experimental batch**			**Dry mass before immersing (g)**	**Dry mass after immersing (g)**	**% Remaining mass**	**Average % Remaining mass**
**Day 0**	Scaffold in FBS	Sample 1	0.0168	0.0179	106.547619	100.1984127
		Sample 2	0.0168	0.0163	97.02380952	
		Sample 3	0.0168	0.0163	97.02380952	
	Scaffold in PBS	Sample 1	0.0139	0.0142	102.1582734	100.2398082
		Sample 2	0.0139	0.014	100.7194245	
		Sample 3	0.0139	0.0136	97.84172662	
**Day 5**	Scaffold in FBS	Sample 1	0.0168	0.018	107.1428571	106.1507937
		Sample 2	0.0168	0.0184	109.5238095	
		Sample 3	0.0168	0.0171	101.7857143	
	Scaffold in PBS	Sample 1	0.0139	0.0144	103.5971223	104.5563549
		Sample 2	0.0139	0.0146	105.0359712	
		Sample 3	0.0139	0.0146	105.0359712	
**Day 10**	Scaffold in FBS	Sample 1	0.0168	0.018	107.1428571	108.7301587
		Sample 2	0.0168	0.0179	106.547619	
		Sample 3	0.0168	0.0189	112.5	
	Scaffold in PBS	Sample 1	0.0139	0.0152	109.352518	106.4748201
		Sample 2	0.0139	0.0148	106.4748201	
		Sample 3	0.0139	0.0144	103.5971223	
**Day 20**	Scaffold in FBS	Sample 1	0.0168	0.0173	102.9761905	104.9603175
		Sample 2	0.0168	0.0175	104.1666667	
		Sample 3	0.0168	0.0181	107.7380952	
	Scaffold in PBS	Sample 1	0.0139	0.0139	100	103.8369305
		Sample 2	0.0139	0.015	107.9136691	
		Sample 3	0.0139	0.0144	103.5971223	

Supplementary Figure

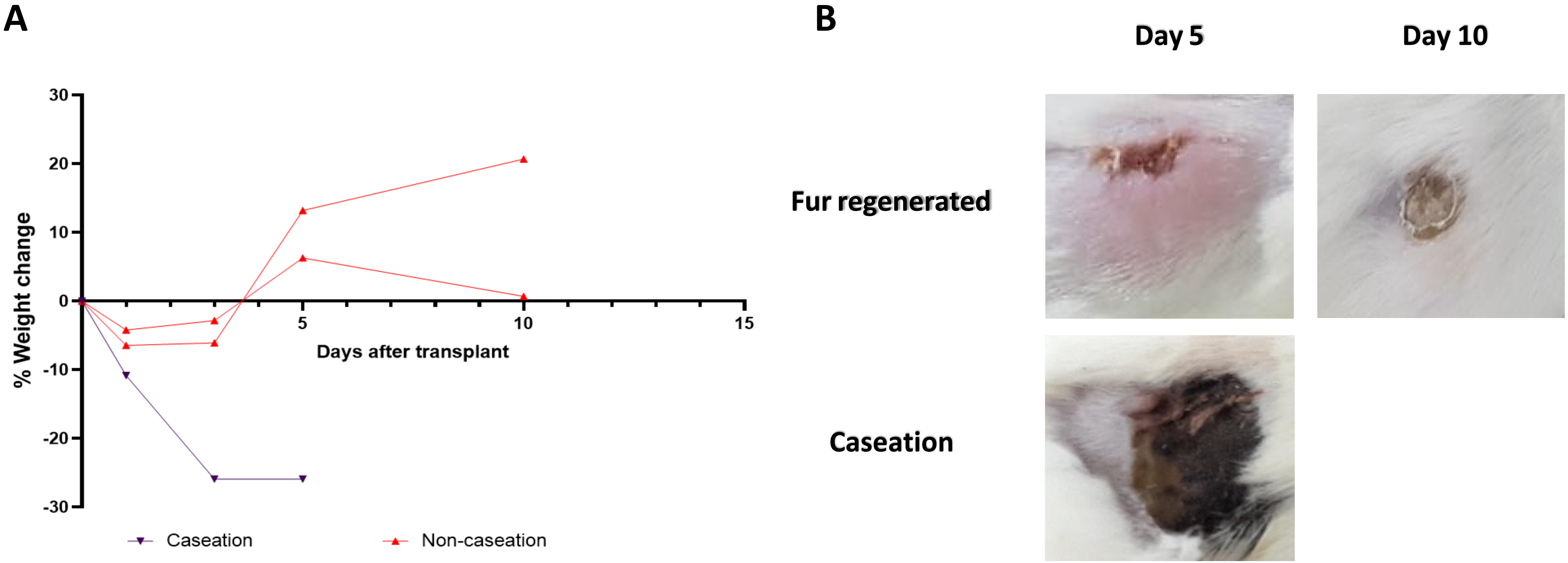

Supplementary Figure S11. (A) % weight change of mice engrafted with porcine tissue. (B) The area above the implant site of mice engrafted with porcine tissue. There were two different outcomes. One mouse was caseatedwith severe weight loss after 5 days, and the others could still regenerate the fur and managed to survive up to day 10.

Supplementary Figure

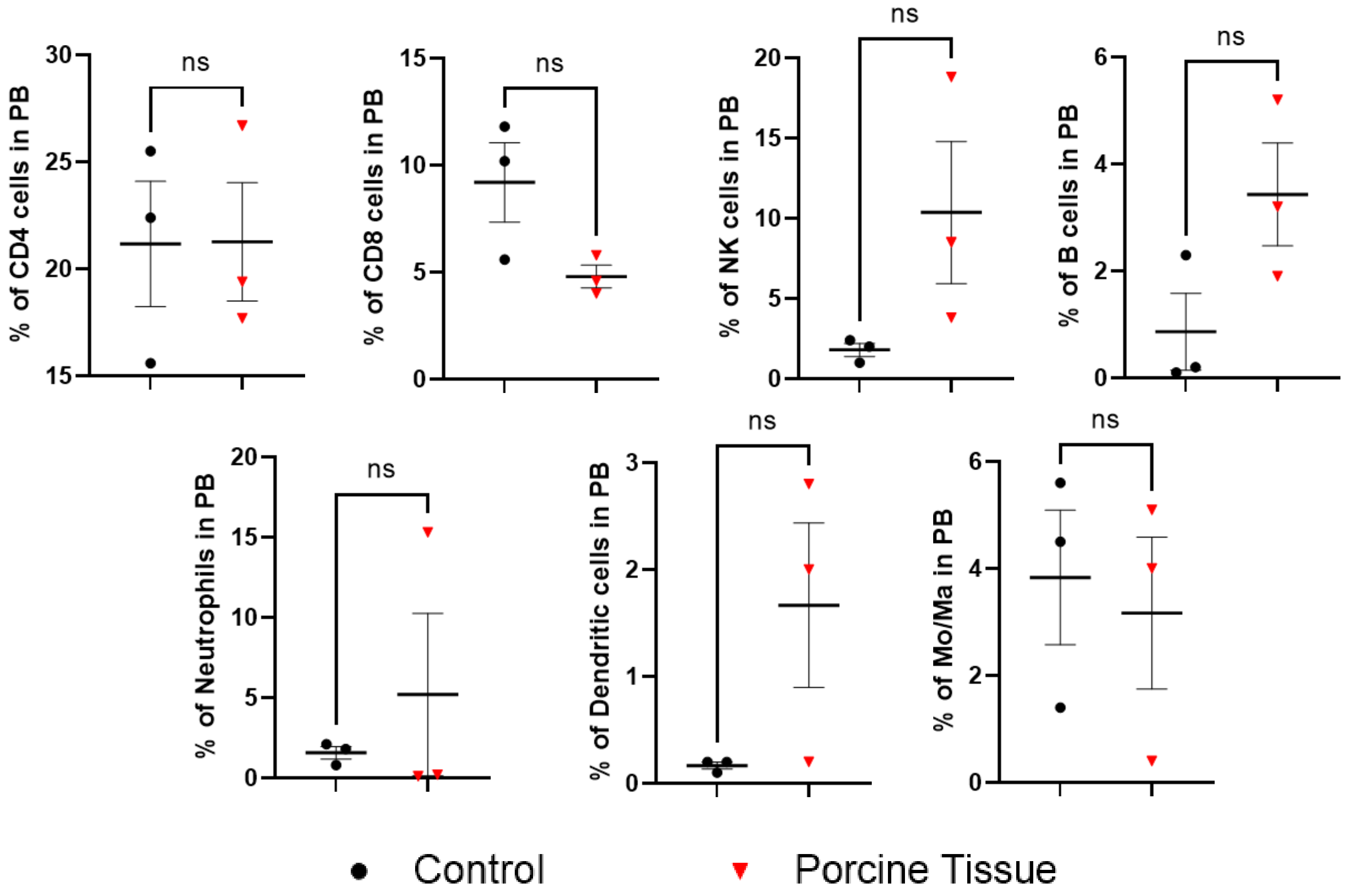

Supplementary Figure S12. Peripheral blood leukocytes analysis of porcine tissue implant group. ns: nonsignificant; **: P ≤ 0.01. No statistical differences were found between the porcine tissue implant group and the control group.

Supplementary Figure

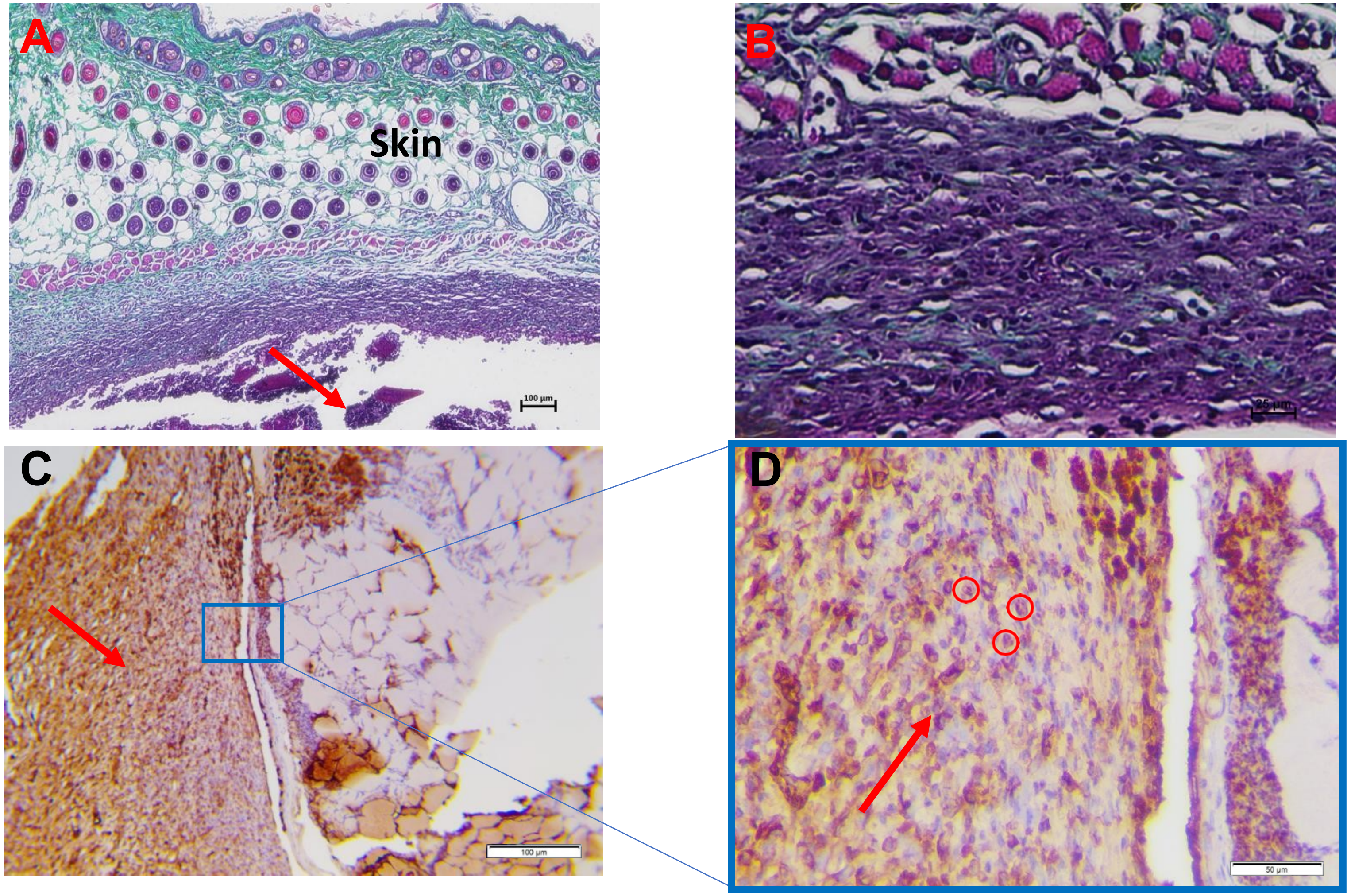

Supplementary Figure S13. Histology staining of mice implanted with porcine tissue. (A)Trichrome staining at 4X magnification, 100µm scale. (B)Trichrome staining at 20X magnification, 25µm scale. (C) Immunohistochemistry staining against hu-CD44 at 10X magnification, 100µm scale. (D) Immunohistochemistry staining against hu-CD44 at 20X magnification, 50µm scale. Red arrow, porcine tissue; red circle, mice leukocytes.
